# Early cytokine signatures and clinical phenotypes discriminate persistent from resolving MRSA bacteremia

**DOI:** 10.1186/s12879-025-10620-3

**Published:** 2025-02-18

**Authors:** Kristina V. Bergersen, Ying Zheng, Maura Rossetti, Felicia Ruffin, Harry Pickering, Rajesh Parmar, Gemalene Sunga, Liana C. Chan, David Gjertson, Vance G. Fowler, Michael R. Yeaman, Elaine F. Reed, the MRSA Systems Immunobiology Group, Kristina V. Bergersen, Ying Zheng, Maura Rossetti, Felicia Ruffin, Harry Pickering, Rajesh Parmar, Liana C. Chan, David Gjertson, Vance G. Fowler, Michael R. Yeaman, Elaine F. Reed, Alexander Hoffmann, Felix Medie, Batu Sharma, Joshua Thaden

**Affiliations:** 1 Department of Pathology and Laboratory Medicine, David Geffen School of Medicine, University of California Los Angeles, 1000 Veteran Ave, Los Angeles, CA 90095 USA; 2 UCLA Immunogenetics Center, Department of Pathology and Laboratory Medicine, David Geffen School of Medicine, University of California Los Angeles, Los Angeles, CA USA; 3 Division of Infectious Diseases, Duke University School of Medicine, 2301 Erwin Road, Durham, NC 27710 USA; 4 Institute for Infection and Immunity, Lundquist Institute at Harbor UCLA Medical Center, Torrance, CA USA; 5 Division of Molecular Medicine, Los Angeles County Harbor-UCLA Medical Center, Torrance, CA USA; 6 Duke Clinical Research Institute, Duke University, Durham, NC USA; 7 Division of Infectious Diseases, Los Angeles County Harbor-UCLA Medical Center, Torrance, CA USA; 8 Divisions of Molecular Medicine and Infectious Diseases, David Geffen School of Medicine and Harbor-UCLA Medical Center, 1124 West Carson Street, Building MRL / 250, Torrance, CA 90502 USA

**Keywords:** *Staphylococcus aureus*, MRSA, Cytokine signatures, Clinical phenotypes, Persistence

## Abstract

**Background:**

*Staphylococcus aureus* bacteremia (SAB) is a prevalent life-threatening infection often caused by methicillin-resistant *S. aureus* (MRSA). Up to 30% of SAB patients fail to clear infection even with gold-standard anti-MRSA antibiotics. This phenomenon is termed antibiotic-persistent MRSA bacteremia (APMB). The mechanisms driving APMB are complex and involve host phenotypes significantly impacting the immune response. Thus, defining early immune signatures and clinical phenotypes that differentiate APMB from antibiotic resolving (AR)MB could aid therapeutic success.

**Methods:**

We assessed 38 circulating cytokines and chemokines using affinity proteomics in 74 matched pairs of vancomycin-treated SAB cases identified as ARMB or APMB after 5 days of blood culture.

**Results:**

Unsupervised hierarchical clustering segregated APMB from ARMB based on differential levels of IL-10, IL-12p40, IL-13, CCL4, and TGFα. Additionally, CXCL1, CCL22 and IL-17A significantly differed between APMB and ARMB when correlated with diabetes, dialysis, metastatic infection, or cardiac vegetation. Combining immune signatures with these relevant clinical phenotypes sharply increased accuracy of discriminating APMB outcome to 79.1% via logistic regression modeling. Finally, classification-regression tree analysis revealed explicit analyte thresholds associated with APMB outcome at presentation especially in patients with metastatic infection.

**Conclusions:**

Collectively, this study identifies previously unrecognized cytokine and chemokine signatures that distinguish APMB and ARMB at presentation and in the context of host clinical characteristics associated with increased disease severity. Validation of a biomarker signature that accurately predicts outcomes could guide early therapeutic strategies and interventions to reduce risks of persistent SAB that are associated with worsened morbidity and mortality.

**Supplementary Information:**

The online version contains supplementary material available at 10.1186/s12879-025-10620-3.

## Introduction

*Staphylococcus aureus* is an etiological agent of both hospital and community infections worldwide [1–3]. Methicillin-resistant *S. aureus* (MRSA) infection often leads to severe *S. aureus* bacteremia (SAB) and is the leading cause of multi-drug-resistant infections in the US [4]. These infections are often associated with poor outcomes including metastatic sequelae, endocarditis, infarction, septic shock, and death [5]. Despite in vitro susceptibility, many SAB isolates fail to clear the bloodstream within 5 days of gold-standard anti-MRSA therapy using vancomycin (VAN) [6–8]. Such infections are termed antibiotic-persistent MRSA bacteremia (APMB) in contrast to antibiotic-resolving MRSA bacteremia (ARMB) that clear upon therapy. Up to 30% of patients experiencing APMB succumb to infection within 3 months of diagnosis [9].

While several patient demographic and clinical phenotypes are commonly associated with more severe MRSA infection [10–16], the impact of these phenotypes on the cytokine and chemokine response to infection has been largely unexplored. While advances in understanding outcomes in SAB have been made using proteomics, transcriptomics, genomics, and epigenomics [17–20], it is currently not possible to predict whether an individual patient diagnosed with SAB would eventuate to APMB or ARMB. Hence, there is a critical, unmet need to identify clinical correlates linking immune signatures and APMB outcomes to develop timely and accurate tools for risk stratification at time of diagnosis. Such a capability could result in prospective improvement of clinical outcomes and mitigate long-term complications. Additionally, such tools may provide mechanistic insights into protective vs. non-protective immunity that may be targetable through vaccine or immunotherapeutic intervention.

In this study, we compared early circulating cytokine and chemokine levels in relation to patient outcome, baseline demographics, and clinical phenotypes associated with more severe MRSA-induced SAB to gain insights into correlates of APMB outcome. Results identified 5 analytes that were significantly increased in expression in APMB, but these analytes alone were not sufficient to identify APMB outcome. However, they displayed dynamic relationships with pertinent clinical phenotypes that were also correlated with APMB, and the analytical combination of immune signatures and clinical phenotypes greatly increased accuracy of identifying APMB outcome in our training cohort. This approach also enabled estimation of relevant cytokine/chemokine threshold values of consequence to clinical outcomes. Thus, advanced modeling suggests the potential for predictive capabilities for APMB outcomes based on specific cytokines and chemokines when paired with relevant clinical parameters.

## Methods

### Patients and sample collection

The primary analysis included a total of 148 eligible adult patients (74 ARMB, 74 APMB) diagnosed with a monomicrobial *S. aureus* bloodstream infection. Patients who died before being approached by a research staff member were enrolled under the IRB Notification of Decedent Research. A subsequent cohort of 108 patients was used for validation studies. Criteria for inclusion in this analysis were: individuals ≥ 18 years of age; culture-positive confirmed MRSA bacteremia; MRSA isolate archived; intravenous vancomycin (VAN) treatment; and clinical metadata available. Exclusion criteria included: MRSA culture-negative or lack of available isolate or clinical metadata; polymicrobial infection; antibiotic therapy other than VAN; neutropenia; outpatients; or those ≤ 18 years of age. Plasma and/or serum samples were collected at time of MRSA infection diagnosis and stored at −80⁰C in the Bloodstream Infections Biorepository (BSIB) at Duke University Medical Center (DUMC). Additionally, research staff collected demographic and clinical data on each enrolled patient and stored the data in an electronic data capture system, i.e. Microsoft Access 2016.

This study was conducted in accordance with Good Clinical Practice and Human Subjects Research as previously approved by the Duke University Medical Center (DUMC) Institutional Review Board (Approval Number: Pro00008031).

### Study design

We conducted a retrospective cohort study of hospitalized patients diagnosed with ARMB or APMB. APMB was defined as ≥ 5 consecutive days of MRSA bacteremia in patients receiving appropriate VAN treatment. Patients classified as APMB with available plasma or serum samples were matched to ARMB patients by stratified propensity score on age, sex, race, and diabetes. The focus on VAN therapy for bacteremia is based on the following cogent factors: (a) VAN was the predominant drug of choice for SAB during the window of patient enrollment in the Duke biorepository cohorts used in this study; (b) it remains a first-line antibiotic for treatment of SAB; and (c) it is less likely to induce inflammatory toxicity than daptomycin, which could conceivably confound immune response data.

### Luminex

Human 38-plex magnetic cytokine/chemokine kits (EMD Millipore, HCYTMAG-60 K-PX38) were used per manufacturer’s instructions on patient plasma samples whenever possible. If plasma samples were not available, serum samples were used. The panel included IL-1RA, IL-10, IL-1α, IL-1β, IL-6, IFN-α2, TNF/TNF-α, TNF-β/LT-α, sCD40L, IL-12p40, IFN-γ, IL-12/IL-12p70, IL-4, IL-5, IL-13, IL-9, IL-17A, GRO/CXCL1, IL-8/CXCL8, Eotaxin/CCL11, MDC/CCL22, fractalkine/CX3CL1, IP-10/CXCL10, MCP-1/CCL2, MCP-3/CCL7, MIP-1α/CCL3, MIP-1β/CCL4, IL-2, IL-7, IL-15, GM-CSF, Flt-3 L/CD135, G-CSF, IL-3, EGF, FGF-2, TGF-α, and VEGF. Fluorescence was quantified using a Luminex 200™ instrument. Cytokine/chemokine concentrations were calculated using Milliplex Analyst software version 4.2 (EMD Millipore). Luminex assay and analysis were performed by the UCLA Immune Assessment Core.

### Statistics and analysis

Descriptive statistics were computed for all study variables, stratifying by patient outcome (APMB or ARMB). Continuous variables (for clinical characteristics and cytokines) were summarized using means and standard deviations, and categorical variables using frequencies and percentages. Continuous and categorical variables were compared between groups using two-way unpaired t-test, Fisher’s exact test, or simple linear regression as appropriate. The threshold for significance was set at *p* = 0.05. Values below the limit of quantitation were put to 0. All analyses of experimental variables were performed after natural log transform of raw data + 1 to obviate concerns of non-normality.

Patterns in expression levels among cytokines, chemokines, and growth factors were analyzed using a variety of clustering approaches. Unsupervised, hierarchical clustering based on Ln-norm dissimilarities and complete-linkage agglomeration were used to create patient and factor dendrograms displayed in heatmaps of expression levels. Multivariate logistic regression models were assessed for fit using receiver operating characteristic (ROC) curves. For correlation analyses, pair-wise Pearson correlations were conducted using the R package *Hmisc*. Correlation analysis results were visualized using the *corrplot* function in R. All continuous variables were scaled from 0 to 1 to guarantee that they were all on the same scale. Categorical variables were converted to 1 (yes) or 0 (no). Correlation coefficients with *p*-values less than 0.05, 0.01, and 0.001 are denoted with “*,” “**,” and “***” respectively. For validation of the logistic regression model on a blinded validation cohort, the “predict” function in STATA was used followed by logistic regression modeling based on the training cohort. CART analysis was performed using the following methods. The *rpart* function in the R library (the R software package version 3.4.0, http://www.r-proje ct.org/) was used to perform a recursive partitioning regression analysis and build a classification tree. In order to avoid over-fitting and to select a parsimonious set of predictor variables, the maximum depth of any node of the final tree (the root node, counted as depth 0) was set to 3, and the minimum number of observations that must exist in a node, in order for a split to be attempted was set to 5. All other parameters were set to their default values.

## Results

### Clinical characteristics of the patient population

Seventy-four subjects meeting inclusion/exclusion criteria were included in the APMB cohort, with 74 ARMB controls on sex, age, race and diabetes status. Table 1 summarizes the demographics and selected clinical characteristics of the cohort. APMB and control ARMB patients had comparable baseline variables of age, sex, race, and diabetes status corresponding with our study design. Looking at clinical measures, APMB patients displayed significantly higher frequencies of metastatic infection (*p* < 0.0001) and longer duration of antibiotics (*p* < 0.0001) compared to ARMB patients, while prevalence of cardiac vegetations seen by trans-tracheal echocardiography (TTE) was comparable between groups.

**Table 1:** Patient demographics and clinical characteristics of the training cohort

Parameter	ARMB (*n* = 74)	APMB (*n* = 74)	*P*-Value
Baseline variables			
Age (yrs)			0.681
Mean (SD)	61.2 (14.02)	60.2 (14.31)	
Sex (%)			1.000
Females	33 (45.2)	33 (45.2)	
Males	40 (54.8)	40 (54.8)	
Race (%)			1.000
Caucasian	37 (50.7)	37 (50.7)	
Black	35 (47.9)	35 (47.9)	
Unknown/other	2 (2.7)	2 (2.7)	
Diabetes (%)			0.476
N	29 (39.7)	34 (46.6)	
Y	43 (58.9)	38 (52.1)	
Hemodialysis (%)			0.074
N	53 (71.6)	43 (58.1)	
Y	21 (28.4)	30 (40.5)	
Clinical measures			
Metastatic Infection (%)			< 0.0001
N	45 (60.8)	16 (21.6)	
Y	29 (39.2)	58 (78.4)	
Duration of antibiotic (days)			< 0.0001
Mean (SD)	29.6 (18.09)	46.9 (22.80)	
Cardiac vegetations by TTE			0.754
N	55 (74.3)	51 (68.9)	
Y	19 (25.7)	23 (31.1)	

### Global cytokine and chemokine expression are differentiated by MRSA infection outcome

We measured circulating levels of 38 cytokines, chemokines, and growth factors at the time of MRSA bacteremia diagnosis using multiplex Luminex bead arrays. Of these analytes, 22 (57.9%) were above the minimum level of detection in at least 75% of patient samples and were included in the analysis (Supplementary Table 1).

**Fig. 1:**
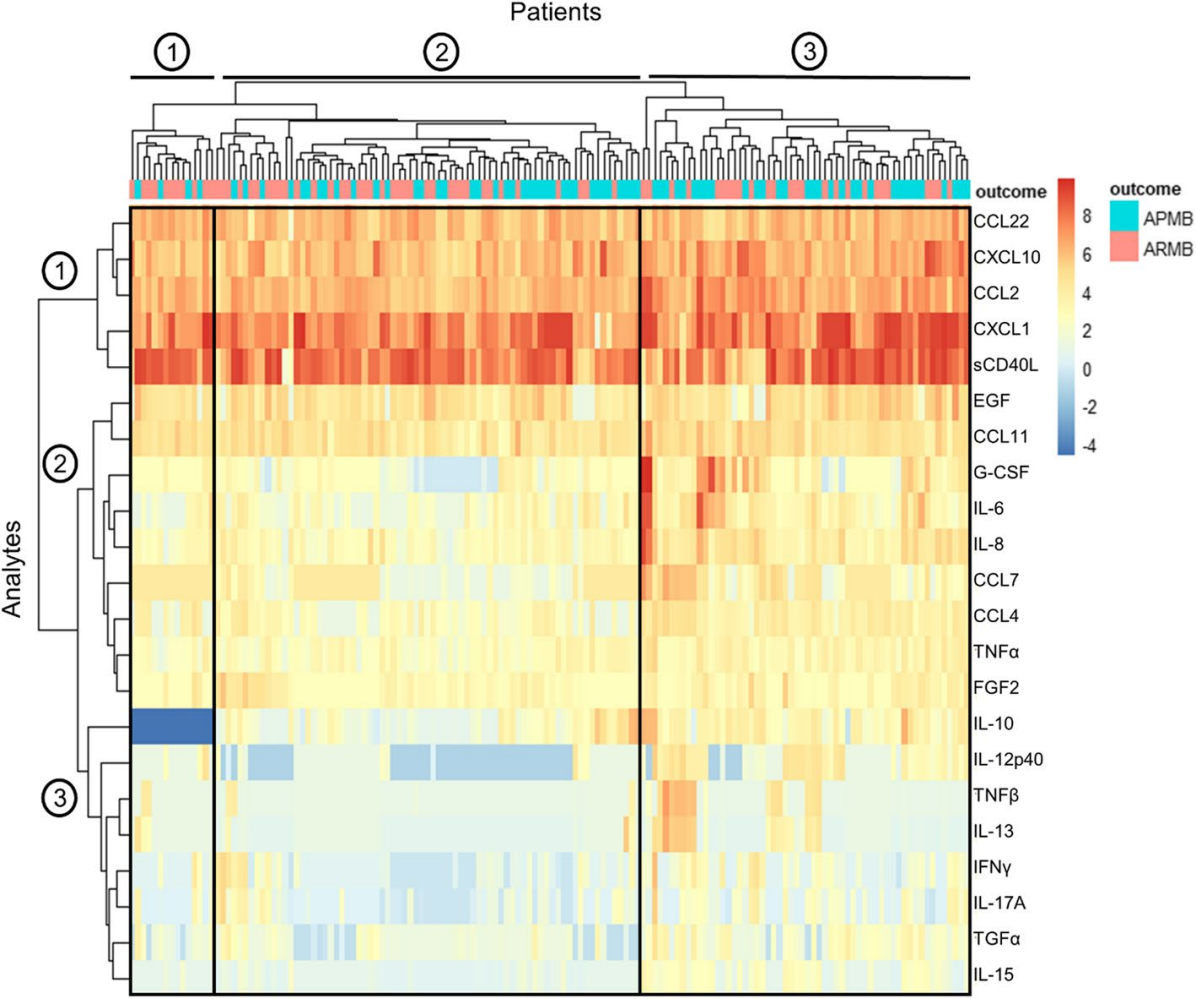
Global cytokine and chemokine expression differentiate MRSA infection outcomes. 38 cytokines, chemokines, and growth factors were measured in peripheral blood of MRSA-infected patients at time of diagnosis by multiplex Luminex bead assay. Heat map shows expression of 22 cytokines that were detected above the minimum level of detection in more than 75% of patient samples and included in the analysis. Columns represent individual patients, rows represent individual cytokines, and colors represent normalized mean cytokine concentration values (blue = low, red = high). Infection outcome classified as “APMB” (turquoise) or “ARMB” (pink). Rows and columns are ordered based on results of unsupervised hierarchical clustering, with dendrograms for the cytokine and patient clusters shown on the horizontal and vertical axes, respectively. Groups 1–3 on X and Y axes represent the main patient clusters (X) and cytokine clusters (Y) as identified by unsupervised hierarchical clustering

We used unsupervised canonical hierarchical clustering [21] to identify proteomic patterns in APMB vs. ARMB patients. The overall patterns of cytokines and chemokines segregated APMB from ARMB patients (Fig. 1). Three major patient clusters and 3 main analyte clusters were identified: (1) patient cluster 1 was composed of mostly ARMB patients and had distinctly reduced IL-10 expression corresponding with the minimum level of detection for this cytokine; (2) patient cluster 2 was composed of an equal amount of ARMB and APMB patients and showed low expression of analyte cluster 3 proteins predominantly involved in Th1 response (IL-12p40 and IFNγ) and immune regulation/Th17 polarization (IL-10, TNFβ, IL-13, IL-17 A, TGFα, IL-15); (3) patient cluster 3 was composed predominantly of APMB patients and showed increased expression of analyte cluster 2 proteins relating to cell survival (G-CSF, CCL7, and CCL4) and early innate immunity (IL-6, IL-8) as well as analyte cluster 3 proteins. Analyte cluster 1 (cell recruitment) proteins were uniformly expressed across all 3 patient clusters. Two chemokines in analyte cluster 2, EGF and CCL11, were also uniformly expressed by all patient clusters independent of infection outcome and are also involved in cell recruitment.

**Fig. 2:**
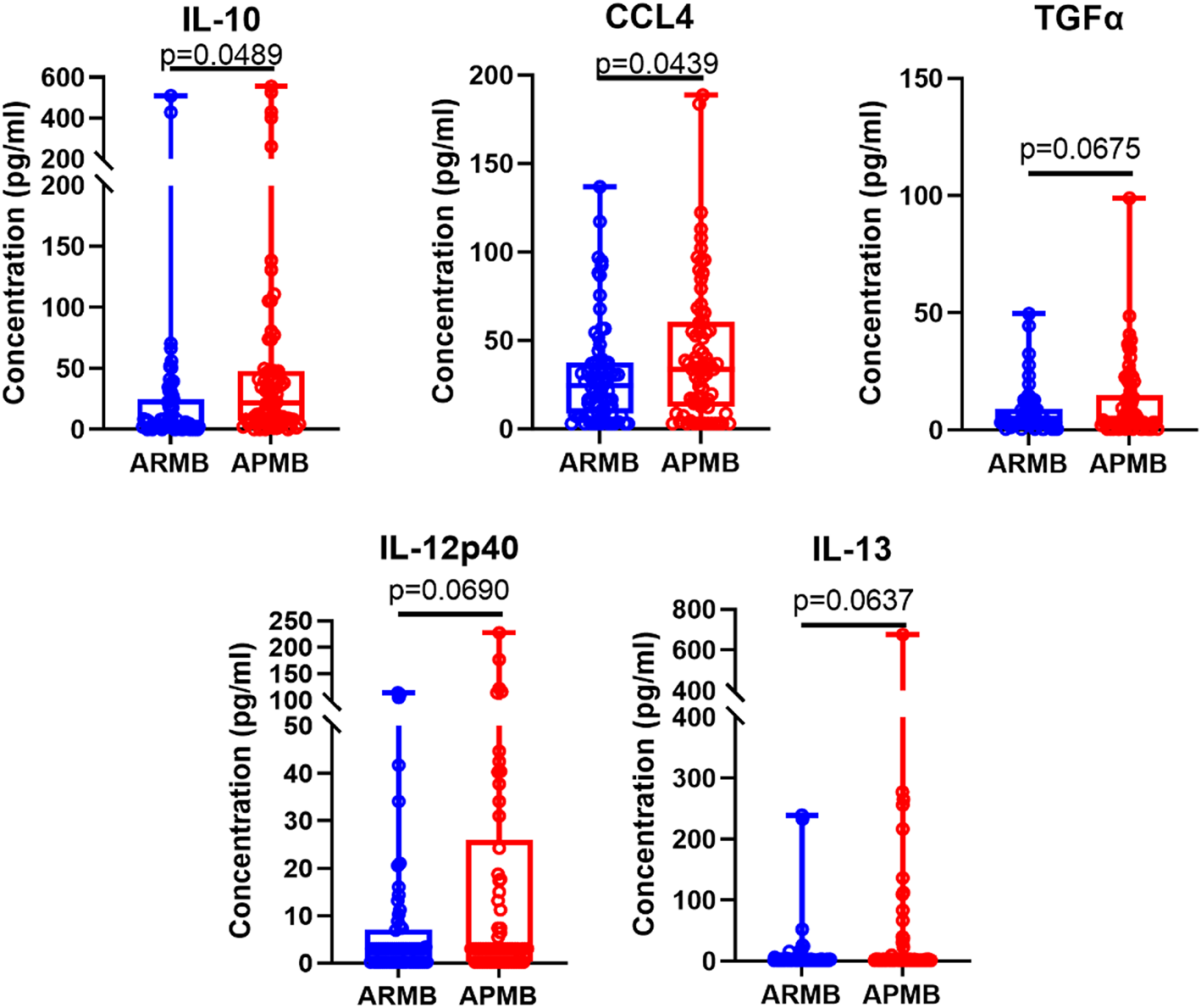
Principal circulating cytokines and chemokines are significantly elevated during persistent infection. Concentrations of five analytes trending (*p* < 0.1) or significantly different (*p* < 0.05) between ARMB and APMB patients in the combined cohort are shown. Significance determined via two-way unpaired t-test

Evaluation of individual analytes for differences between APMB and ARMB patients revealed that the mean concentrations of circulating IL-10 and CCL4 were significantly elevated in APMB patients, while TGFα, IL-12p40, and IL-13 all displayed strong trends of elevation in APMB vs. ARMB patients (Fig. 2). These results suggest that early immune responses to MRSA bacteremia evoke distinct signatures that contribute to differential APMB vs. ARMB outcomes.

### Early principal cytokine and chemokine levels alone are not sufficient to discriminate APMB outcome

**Fig. 3:**
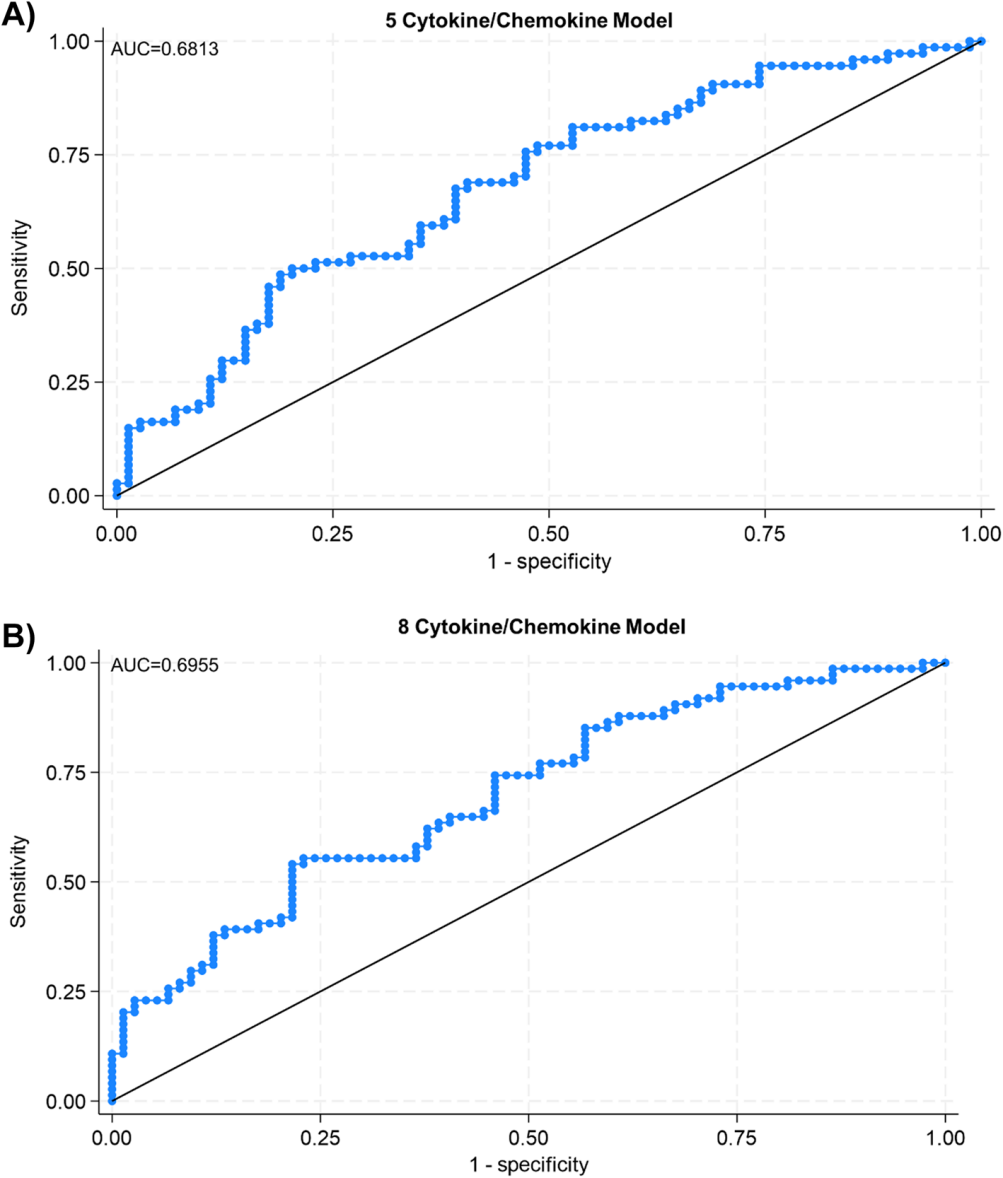
Principal cytokines chemokines alone are insufficient to discriminate APMB outcome. **A** Logistic regression model for combined training cohort. ROC curve built using normalized (ln) values of 5 cytokines shown in Fig. 2. **B** Logistic regression model for combined training cohort. ROC curve built using normalized (ln) values of 5 cytokines shown in Figs. 2 and 3 additional cytokines (CXCL1, CCL22, IL-17A) identified by Lasso regression

We next explored the capability of the 5 APMB-elevated analytes alone to discriminate APMB outcome in our training cohort. To accomplish this, we utilized a logistic regression model to assess the predictive value of these analytes independent of any clinical phenotypes or outcomes. The accuracy of the 5 analytes in differentiating APMB from ARMB, represented by the area under the curve (AUC) for the logistic regression ROC curve, was 68.1% (Fig. 3A). This suggests that although IL-10, CCL4, TGFα, IL-12p40, and IL-13 are elevated in APMB compared to ARMB, their composite immune signature alone is insufficient to accurately discriminate APMB outcome.

Because of the striking separation seen in global cytokine and chemokine expression between APMB and ARMB patients, we next used all 22 analytes above the limit of detection as input for a Lasso regression model to determine whether there were any additional molecules that could aid in discrimination. Lasso regression identified 6 analytes as having discriminative capability: CXCL1, IL-10, CCL22, IL-17A, IL-12p40, and IL-13 (Supplemental Fig. 1). Addition of CXCL1, CCL22, and IL-17A to the original 5 APMB-associated analyte model did not significantly increase accuracy (69.6%, Fig. 3B), nor did use of the 6 Lasso-identified analytes by themselves (data not shown). These results collectively indicate that circulating cytokine and chemokine levels alone are not sufficient to accurately discriminate APMB from ARMB. Thus, other phenotypes must be considered when discriminating persistent vs. resolving MRSA bacteremia outcomes.

### APMB-specific relationships exist between early immune signatures and clinical phenotypes

**Fig. 4:**
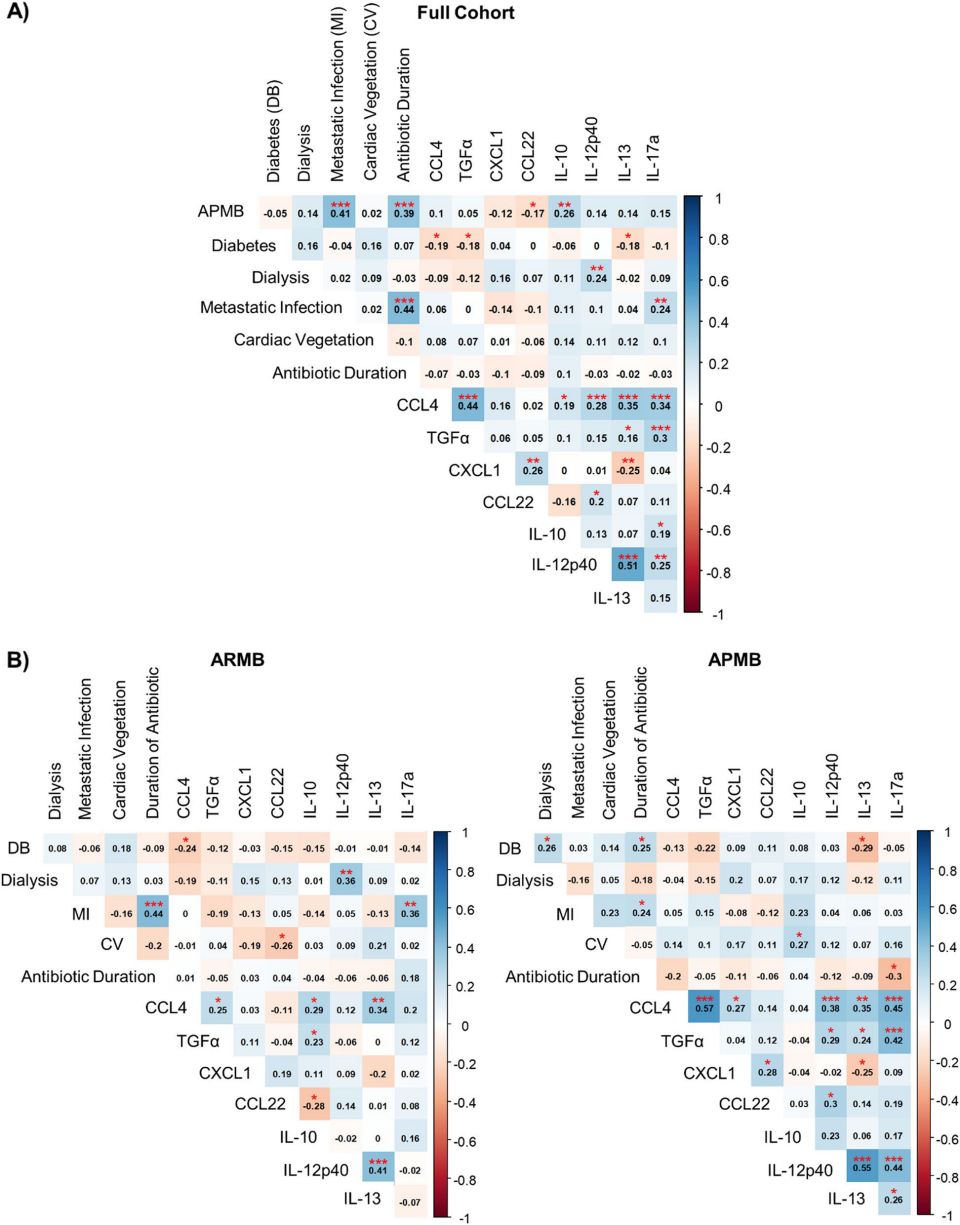
Outcome of MRSA infection is correlated with the interactions between circulating analytes and clinical phenotypes. Pearson correlation analyses were performed to evaluate the relationship between original and Lasso-identified predictive cytokines and chemokines and selected clinical variables. Correlations are shown from strongly negative (dark red) to strongly positive (dark blue) between individual cytokines and chemokines (normalized (ln) values) and clinical variables. Strength of correlation is shown by darkness of box. Numbers in individual boxes show correlation value between 2 parameters. Any significant correlations between parameters are marked with the following to demonstrate level of significance: * = *p* < 0.05, ** = *p* < 0.01, *** = *p* < 0.001. **A** Correlation matrix of cytokines and clinical variables in full combined cohort. **B**, **C** Additional correlation matrices separated by ARMB (**B**) vs. APMB (**C**) infection outcome

To investigate the unexplored interplay between early immune signatures and clinical phenotypes for potentially targetable differences between APMB and ARMB, we performed global Pearson correlation analyses using the 8 cytokines and chemokines identified above in composite with pertinent clinical phenotypes associated with more severe MRSA-induced SAB. In the full cohort, APMB outcome was significantly positively correlated with the presence of metastatic infection, prolonged antibiotic duration, and increased circulating IL-10, and was significantly negatively correlated with elevated CCL22 (Fig. 4A). When the cohort was stratified by outcome, ARMB patients had more negative correlations between analytes and clinical phenotypes than APMB as expected (Fig. 4B, left). Significant positive correlations that were ARMB-specific were observed between dialysis dependence and elevated IL-12p40, and presence of metastatic infection with elevated IL-17A. In addition, increased CCL22 was significantly negatively correlated with elevated IL-10 in ARMB. In the APMB group, the majority of analytes having significant differences in levels between APMB and ARMB were positively correlated with each other (Fig. 4B, right). APMB-specific significant positive correlations existed between the co-presence of diabetes and dialysis dependence, presence of diabetes and longer antibiotic duration, and presence of cardiac vegetation and elevated IL-10. Metastatic infection was positively correlated with increased IL-10 that trended towards significance in APMB compared to a negative correlation in ARMB. These results collectively implicate targetable outcome-specific relationships between immune and clinical signatures, with the majority of these being significantly associated with persistence.

### Cytokine and chemokine levels in APMB are differentially correlated with individual co-morbidities and disease sequelae

To determine if and how cytokine and chemokine signatures in APMB patients are related to individual clinical phenotypes, we evaluated analyte levels based on the presence or absence of underlying co-morbidities and disease sequelae. Within the APMB group, correlation analyses demonstrated that the demographic parameters of race, sex, or age did not significantly impact any of the 5 APMB-associated analytes (Supplemental Fig. 2). Corresponding to our global correlation analyses, APMB patients with diabetes had lower levels of IL-13 and trended toward decreased CCL4 as compared to those who did not (Supplemental Fig. 3A). Dialysis dependence had no significant correlation with APMB-associated analyte levels (Supplemental Fig. 3B). These results indicate that when analyzed individually, the presence of diabetes or dialysis is not associated with the elevation of cytokines and chemokines observed in APMB.

To determine the relationship between individual SAB-associated disease sequelae and APMB-associated cytokines and chemokines, levels of these proteins in APMB patients were analyzed based on the presence of metastatic infection, cardiac vegetation, and overall mortality. Circulating IL-10 was increased in APMB patients presenting with metastatic infection and cardiac vegetation (Supplemental Fig. 4A-B). Separately, this result further corresponded to significantly elevated IL-10 in APMB patients that succumbed to infection (Supplemental Fig. 4C). APMB-attributable mortality also correlated with significantly elevated TGFα and IL-12p40, but this elevation was independent from metastatic infection or cardiac vegetation. Taken together, these results indicate a significant association between individual disease sequelae and elevated IL-10 seen during APMB-associated mortality, but these phenotypes do not correlate with the other elevated analytes seen in APMB-associated mortality.

### Composite clinical and immune signatures increase accuracy to discriminate APMB outcome and mortality

**Fig. 5:**
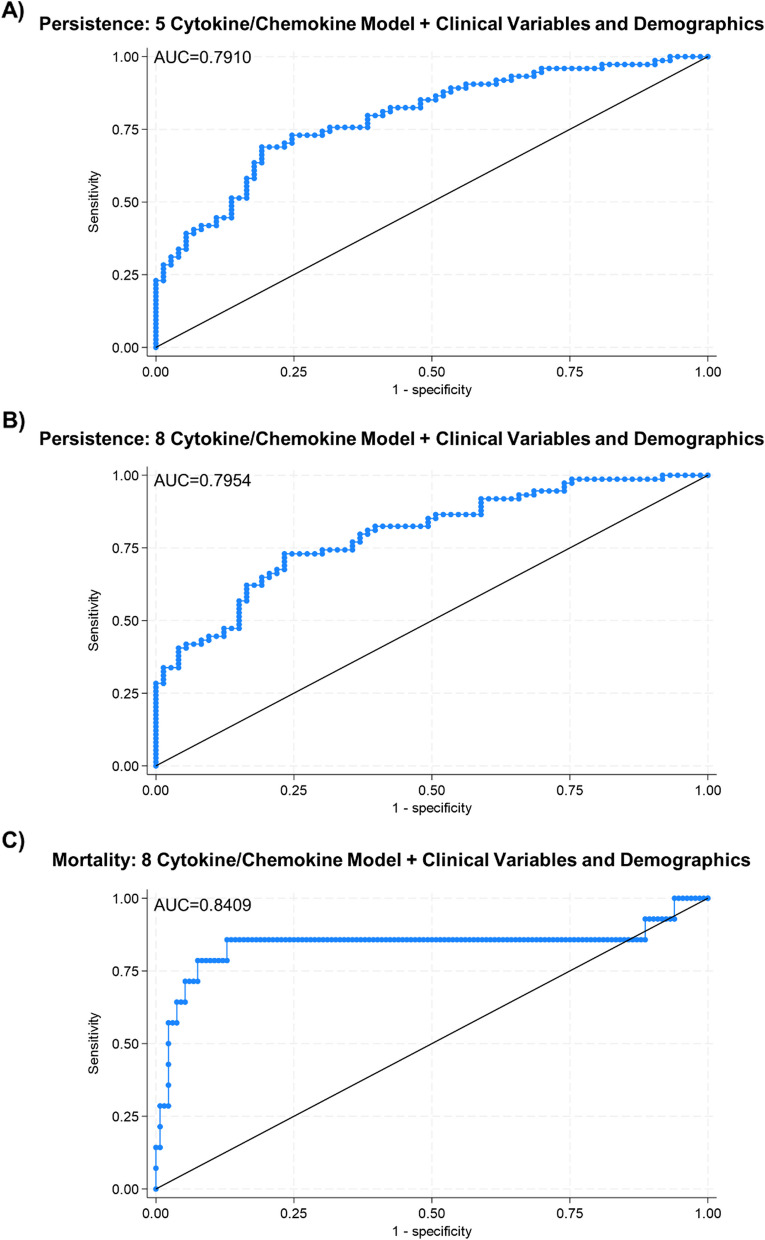
Inclusion of clinical phenotypes increases accuracy of discriminating APMB outcome and mortality. **A** Logistic regression model predicting persistence for training cohort. ROC curve built using normalized (ln) values of 5 cytokines/chemokines and 4 selected clinical variables. **B** Logistic regression model predicting persistence for training cohort. ROC curve built using normalized (ln) values of 8 cytokines/chemokines and 4 selected clinical variables. **C** Logistic regression model predicting mortality for training cohort. ROC curve generated using normalized (ln) values of 8 selected cytokines/chemokines and 4 selected clinical variables

The correlations of clinical parameters with APMB vs. ARMB outcome and specific immune signatures led us to hypothesize that addition of clinical phenotypes as independent variables to our analyte model would increase accuracy in discriminating APMB. We first investigated the accuracy of clinical phenotypes alone in identifying APMB outcome in the training cohort. Use of the 4 pertinent clinical phenotypes (diabetes, dialysis dependence, metastatic infection, and cardiac vegetation) in a logistic regression model yielded an AUC of 75.2% (Supplemental Fig. 5). Addition of these 4 clinical phenotypes plus the demographic parameters of race, age, and sex to our original 5 APMB-associated cytokine/chemokine model further increased the AUC to 79.1% (Fig. 5A). In this model, IL-10 and metastatic infection were the phenotypes that most significantly influenced accuracy in support of our previous results. Logistic regression modeling combining the 8 cytokines/chemokines, all 4 clinical phenotypes, and demographic parameters led to the highest AUC of 79.6% (Fig. 5B). Analysis of the 8-cytokine/chemokine model with single clinical phenotypes revealed metastatic infection (79.0% AUC) as the strongest individual clinical phenotype in discriminating persistence when paired with immune signatures (Supplemental Fig. 6). Use of this same model to accurately identify mortality demonstrated an AUC of 84.1% with IL-17A as the most influential factor (Fig. 5C).These results collectively demonstrate the pertinence of combining immune signatures with relevant clinical phenotypes to accurately discriminate an outcome of APMB during early MRSA bacteremia, especially in individuals experiencing MRSA bacteremia resulting from metastatic infection.

### Composite clinical and immune signatures predict APMB outcome in an independent masked validation cohort

To validate our findings from our training cohort, we applied the same analytical pipeline to an independent masked cohort of 108 patients with ARMB (*n* = 71) or APMB (*n* = 37). Of these individuals, 82 patients (ARMB = 51, APMB = 31) had data available for all 4 clinical phenotypes (Table 2) and were included in logistic regression analysis to predict outcome. Our 8 cytokine/chemokine + 4 clinical phenotype model yielded a 62.0% accuracy overall in predicting outcome (APMB or ARMB) in this masked cohort (Table 3). Pertaining specifically to APMB outcome, this model showed a sensitivity of 64.0%, specificity of 84.1%, and an ROC of 0.697. Due to the high number of patients in the blinded cohort with missing data in the field of cardiac vegetation (Table 2, *n* = 26 (24.2%)) and low overall number of patients with cardiac vegetation present (Table 2, *n* = 7 (6.5%)), we also tested our model without the inclusion of the “cardiac vegetation” phenotype.

**Table 2:** Patient demographics and clinical characteristics of the independent masked validation cohort

Parameter	ARMB (*n* = 71)	APMB (*n* = 37)	*P*-Value
Baseline variables			
Age (yrs)			0.750
Mean (SD)	61.9 (17.27)	63.0(17.60)	
Sex (%)			0.690
Females	31 (43.7)	14 (37.8)	
Males	40 (56.3)	23 (62.2)	
Race (%)			0.013
Caucasian	50 (70.4)	20 (54.1)	
Black	21 (29.6)	13 (35.1)	
Unknown/other	0 (0)	4 (10.8)	
Diabetes (%)			0.049
N	44 (62.0)	15 (40.5)	
Y	26 (36.6)	22 (59.5)	
Unknown	1 (1.4)	0 (0)	
Hemodialysis (%)			0.020
N	65 (91.6)	27 (73.0)	
Y	6 (8.4)	10 (27.0)	
Clinical measures			
Metastatic Infection (%)			0.0002
N	47 (66.2)	10 (27.0)	
Y	24 (33.8)	27 (703.0)	
Duration antibiotic (days)			0.0001
Mean (SD)	35.3 (17.72)	53.1 (28.38)	
Cardiac vegetations by TTE			0.221
N	48 (67.6)	27 (73.0)	
Y Unknown	3 (4.2) 20 (28.2)	4 (10.8) 6 (16.2)	

**Table 3:** Prediction modeling for validation cohort using original model for plasma and serum samples^a^

Parameter	ARMB or APMB (*n* = 82)	ARMB (*n* = 51)	APMB (*n* = 31)
Outcomes Accurately Predicted	51	37	14
Sensitivity (%)	45.2	27.3	64.0
Specificity (%)	72.5	58.3	84.1
ROC Area	0.589	0.480	0.697
Likelihood ratio (+)	1.65	0.911	2.97
Likelihood ratio (-)	0.756	0.527	1.08
Odds Ratio	2.18	0.862	5.50
Positive Predictive Value (%)	62.2	47.7	74.8
Negative Predictive Value (%)	57.0	48.0	65.5
Overall Prediction Accuracy = (37 + 14)/82 = 62%			

^a^Values determined using 95% confidence interval

The 8 cytokine/chemokine + 3 clinical phenoptype model increased overall prediction accuracy to 65.0%, with a sensitivity of 67.6%, specificity of 83.4%, and ROC of 0.717 pertaining specifically to APMB outcome (Table 4).

**Table 4:** Prediction modeling for validation cohort, without cardiac vegetation parameter, for plasma and serum samples^a^

Parameter	ARMB and APMB (*n* = 97)	ARMB Only (*n* = 63)	APMB Only (*n* = 34)
Outcomes Accurately Predicted	63	46	17
Sensitivity (%)	50.0	32.4	67.6
Specificity (%)	73.0	60.3	83.4
ROC Area	0.615	0.513	0.717
Likelihood ratio (+)	1.85	1.09	3.14
Likelihood ratio (-)	0.685	0.474	0.990
Odds Ratio	2.71	1.14	6.43
Positive Predictive Value (%)	64.9	52.2	75.8
Negative Predictive Value (%)	59.4	50.3	67.8
Overall Prediction Accuracy = (46 + 17)/97 = 65%			

^a^Values determined using 95% confidence interval

Because our blinded cohort also contained a substantial number of serum samples compared to our training cohort, we also tested our modified 8 cytokine/chemokine + 3 clinical phenotype model (missing cardiac vegetation parameter) on plasma vs. serum samples to determine whether this model performed better with one sample type over another. Prediction results using plasma samples only yielded an overall accuracy of 71.0%, with an APMB-specific sensitivity of 90.0%, specificity of 82.0%, and ROC of 0.832 (Supplementary Table 2). This was greatly improved over the use of our model with serum samples only (Supplementary Table 3; Overall Accuracy = 60.0%, APMB Sensitivity = 35.0%, APMB Specificity = 96.0%, APMB ROC = 0.683). Taken together, these results validate our findings from our training cohort and demonstrate higher efficacy of our model using plasma samples.

### Classification-regression tree analysis reveals explicit analyte thresholds associated with APMB outcome

**Fig. 6:**
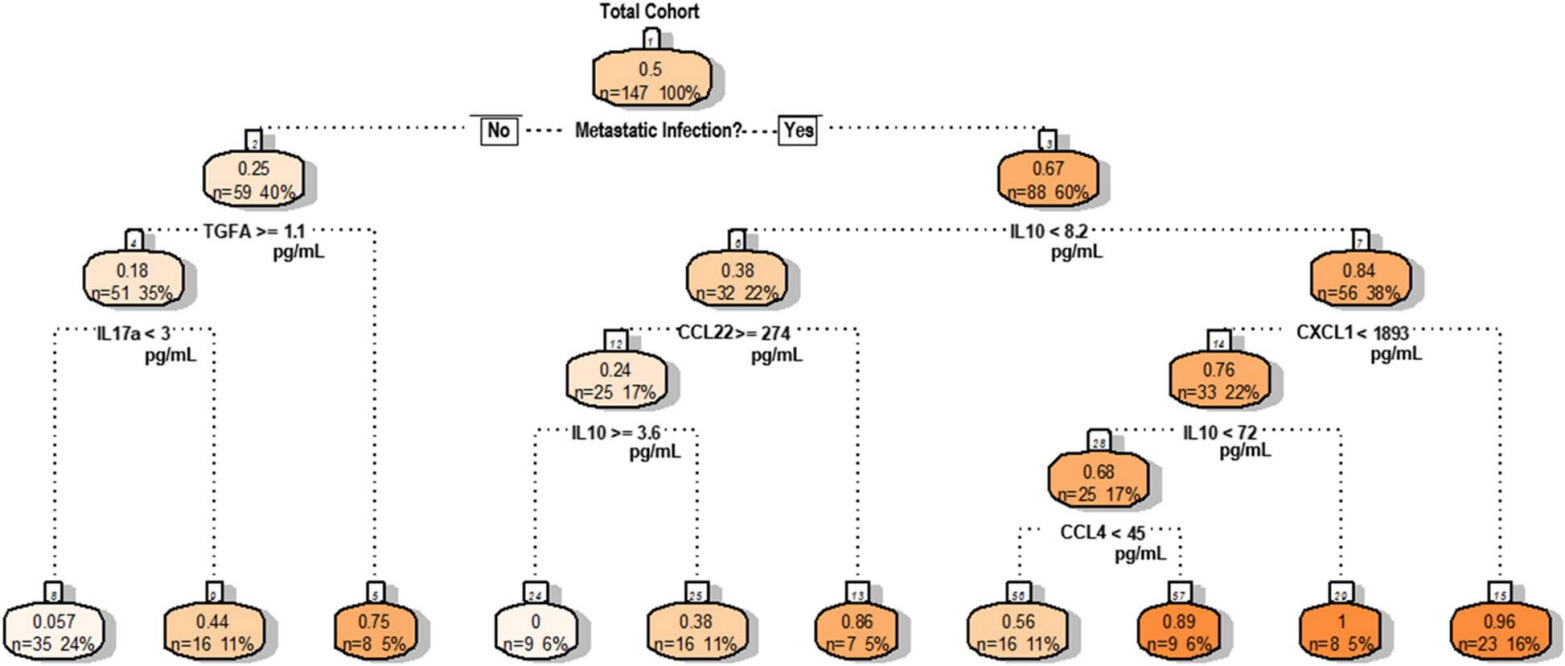
Classification-regression tree (CART) analysis of persistence parameters. CART analysis was performed for 8 cytokines/chemokines and 4 clinical phenotypes from logistic regression modeling to determine analyte thresholds associated with APMB outcome. “Yes” answers to threshold limits (ex. IL10 < 8.2pg/mL) branch left from title while “No” answers to threshold limits (i.e. IL10 > 8.2pg/mL) branch right. Cytokine/chemokine thresholds determining persistent outcome are shown in raw pg/mL values. Some analytes (i.e.IL-10) appear multiple times, and their thresholds may change based on previous nodes (see figure for specific threshold values)

Finally, we performed classification-regression tree (CART) analysis of the 8 cytokines/chemokines (IL-10, CCL4, TGFα, IL-12p40, IL-13, CXCL1, CCL22, and IL-17A) and 4 orignial clinical phenotypes (diabetes, dialysis, metastatic infection, and cardiac vegetation) in our training cohort to determine clinically relevant analyte thresholds that could correctly discriminate a later APMB outcome at presentation of MRSA bacteremia. This analysis revealed distinct circulating cytokine/chemokine thresholds that corresponded to development of APMB and differed greatly depending on the presence or absence of metastatic infection (Fig. 6). From the total cohort, 88 out of 147 patients (60.0%) had metastatic infection, 67.0% (0.67 value) of which were identified as APMB outcome. Within this group, a higher likelihood of APMB outcome (darker orange shades) was identified in patients with IL-10 levels > 8.2pg/mL and CXCL1 levels > 1.89ng/mL (1893pg/mL). If CXCL1 was < 1.89ng/mL, then IL-10 levels > 72pg/mL indicated a higher likelihood of APMB outccome. Finally, if CXCL1 was < 1.89ng/mL and IL-10 level < 72pg/mL, then CCL4 > 45pg/mL correlated with an APMB outcome. There was a small cohort of patients with metastatic infection and IL-10 < 8.2pg/mL that indicated a stronger likelihood of APMB outcome, corresponding with low CCL22 levels (< 274pg/mL). Notably, there was also a small percentage of patients that did not have metastatic infection that developed APMB as determined by very low circulating TGFα (< 1.1pg/mL). In total, this CART analysis was able to accurately identify 55 out of 74 (74.32%) patients with APMB outcome in our training cohort. These results identify a distinct cytokine/chemokine profile with clinically relevant thresholds that correlate with APMB outcome especially in patients with metastatic infection.

## Discussion

### Study significance and summary

Patients who present with SAB due to MRSA and are treated with VAN eventually have either APMB or ARMB outcomes. To date, these outcomes have been clinically indistinguishable at the time of diagnosis, revealing a previously unmet need to determine early discriminators of APMB and ARMB and early predictors of persistence. Previous work by our group and others has investigated the association between pathogenic MRSA phenotype and patient outcome of SAB, identifying several in vitro correlates that could help distinguish between APMB and ARMB. On the pathogen side, such determinants include biofilm formation, *agr* dysfunction, and reduced susceptibility to host defense cationic peptides [7, 22–26]. On the host side, our previous discovery of a polymorphism in the DNA methyltransferase 3A (*DNMT3A*) gene associated with APMB and increased IL-10 levels support the concept of epigenetic determinants of MRSA bacteremia outcomes [27, 28]. Other aspects of host immune response associated with APMB or ARMB outcomes include IL-17A and mature neutrophil response [28], as well as T and B cell activation [20]. Predictors of early and late mortality have also been reported in relation to MRSA bacteremia, including age, presence of certain co-morbidities, and source of bacteremia [17–19].

Recently, advances have been made in understanding differences in the transcriptional immune landscape between APMB and ARMB. For example, APMB outcomes correlate with increased circulating IL-10 and increased levels of immature neutrophils [20, 28]. However, our understanding of global cytokine and chemokine responses that differ between APMB and ARMB, specifically how IL-10 relates to other cytokines or chemokines with respect to severity-associated clinical phenotypes, is incomplete. In this study, we have identified a group of APMB-specific analytes (IL-10, CXCL1, IL-12p40, IL-13, CCL22, CCL4, IL-17A, and TGFα) that correlates with several clinical phenotypes associated with more severe SAB (diabetes, dialysis dependence, metastatic infection, and cardiac vegetation). These immune and clinical phenotypes display dynamic relationships with each other that successfully discriminate APMB from ARMB when combined. We also demonstrate clinically relevant analyte thresholds via CART analysis that can be used to stratify risk of APMB development at time of MRSA bacteremia presentation, especially in patients at increased risk of complicated MRSA infection.

### Dysregulated cytokine and chemokine response in APMB

Evaluation of cytokine and chemokine responses early in the course of SAB is clinically relevant as it could guide therapeutic decisions and potentially improve outcomes. The production of cytokines and chemokines is vital for optimal coordination of innate and adaptive immune responses to infection. Protective immune responses to MRSA include synergistic Th1 and Th17 programs mediated by hallmark cytokines and chemokines [29, 30]. These responses include CD4+ T cell polarization to Th17 paradigm and IL-17A expression. In turn, IL-17A induces CXCL8 and other chemokines to recruit and activate neutrophils at sites of infection. In parallel, IFNγ expression by Th1-polarized CD4+ T cells activates complementary cell-mediated effector responses to aid in MRSA clearance. Subsequent production of IL-6, IL-12, and CXCL1 by neutrophils recruit additional adaptive cells to the site of infection. Collectively, these responses amplify neutrophil-mediated microbicidal killing of MRSA.

The regulatory cytokine IL-10 has also been extensively studied in the context of SAB, and several reports have demonstrated increased IL-10 levels as associated with worsened SAB outcomes. A clear association between increased IL-10 levels at hospital admission and SAB-related mortality has previously been reported [31–34]. Additional work has reported an association between sustained elevation of circulating IL-10 at time of admission and persistence; however, persistence was only able to be predicted through combined IL-10/TNF-α ratios [35]. While associations have been shown between SAB persistence and elevated IL-10 and TNF quartile ranges [30], this relationship was observed predominantly in MSSA infection and showed no significance in aiding prediction outcomes in bacteremia due to MRSA [33]. In a previous case-control study, our group identified IL-10 as one of the chief biomarkers associated with APMB among patients lacking a gain-in-function DNMT3A genotype [36]. More recently, Parmar et al. [20] and Chin et al. [27] separately demonstrated the ability of IL-10 levels to predict MB persistence at the time of diagnosis when paired with genotypic and transcriptomic phenotypes using this same study cohort. These studies and our current findings support the use of IL-10 as one of the main early biomarkers in determining whether MRSA bacteremia will eventuate to APMB. This cytokine may also be implicated in the immunopathology of persistence itself. IL-10 most commonly acts as an immunomodulatory cytokine influencing both innate and adaptive immune responses and could shape the mixed Th1/Th17 response that is largely considered to be responsible for protective immunity against *S. aureus* infection [29, 37–43]. Further, *S. aureus* can trigger IL-10 production in monocytes and macrophages as well as B1a regulatory cells, which in turn may dampen Th1/Th17 responses and induce FOXP3 expression, emblematic of regulatory T cells [44–49]. The increase seen in IL-10 levels in APMB patients in our study and its predictive capability indicates a major dysregulation of the normal cytokine response needed for coordinated protective immunity to clear SAB.

Additional analytes identified in our study that were significantly associated with APMB outcome and suggest a dysregulated cytokine/chemokine response include CXCL1, IL-12p40, IL-13, CCL22, IL-17A, CCL4, and TGFα. Many of these analytes have been identified previously as important in immune response to MRSA infection in some context. While elevated CXCL1 has been previously associated with *S. aureus* bloodstream infection [50], its role in development of persistent MRSA bacteremia has not been previously studied. It is known that this chemokine is closely associated with neutrophil-mediated microbicidal killing of pathogens [51]. The identification of CXCL1 via Lasso regression as an important chemokine in predicting APMB outcome is consistent with two possibilities regarding the pathophysiological development of APMB: (1) compensatory upregulation of CXCL1 in an attempt to stimulate poor microbicidal efficacy of abundant immature neutrophils as observed in our prior studies [27]; and/or (2) impaired neutrophil killing of MRSA. IL-12 has been previously identified as a key cytokine necessary for clearance of MRSA infection [52]. The elevation of IL-12p40 in the current APMB cohort suggests continued over-production of this cytokine by neutrophils and T cells in response to MRSA infection. However, this response is insufficient to avert eventual APMB infection, consistent with prior findings [20, 27]. IL-13 is a Th2-associated cytokine that modulates potentially harmful Th1-polarized responses, such as excess IFNγ production by T cells during MRSA infection. IFNγ is essential for activation of macrophages for intracellular killing of *S. aureus* but is modulated by production of IL-13 and other cytokines to prevent excess inflammation during response to infection. We postulate that the elevation of IL-13 seen in APMB as compared to ARMB patients, paired with no change in circulating IFNγ, reflects either host dysregulation in Th2 response, potential immune evasion by MRSA, or both effects which consequently lead to APMB. This hypothesis is consistent with the findings of Parmar et al. that demonstrate dysregulated T cell transcriptional signatures in APMB [20]. Previous work has demonstrated a vital role of IL-17A in clearance of MRSA in skin and soft tissue infections via Th17 cell responses [43, 53]. However, in the context of bacteremia, recent work has found that elevated IL-17A is correlated with APMB and mortality, especially when paired with IL-10 levels [36, 54–56]. Our results demonstrate circulating IL-17A as the most influential cytokine in predicting mortality from APMB and support these findings, suggesting subversion of IL-17A in host response to bacteremia by persistent MRSA isolates. This concept is strongly supported by the recent findings of Hajam et al., suggesting Th2 and Th10 subversion of immune responses to MRSA are responsible for poor outcomes in MRSA vaccine development [57].

Our findings also identified TGFα, CCL4, and CCL22 as important early discriminators of APMB in the context of associated clinical phenotypes. These associations with APMB have not been previously reported. Specifically, in the APMB cohort, these three analytes were significantly and positively correlated with each other as well as IL-12p40, IL-13, and IL-17A that have a well-known role in MRSA infection. We hypothesize that this pattern of cytokines reflects a potential positive feedback loop during MRSA bacteremia that biases toward non-canonical cytokine/chemokine responses that are permissive to MRSA adaptations, resulting in APMB. Taken together, the APMB-associated cytokines and chemokines identified in this study suggest dysregulation of canonical cytokine and chemokine responses in APMB favoring dissemination of MRSA bacteria, deeper seeding of infection, and development of APMB and indicate a composite cytokine signature, not just IL-10, as a possible tool in the discrimination of APMB from ARMB at time of diagnosis.

### Impact of clinical phenotypes on immune response and outcome

The current study utilized Lasso regression to discover that CXCL1, CCL22, and IL-17A levels in context of specific clinical phenotypes differentiate APMB from ARMB, despite uniform expression of these analytes seen in SAB patient outcomes. These results suggest a complex, APMB-specific interplay between host cytokines/chemokines and the presence of certain clinical phenotypes. While many prior studies often focus on single variable relationships to determine direct cause-and-effect mechanisms and limit variability, outcomes of infection are complex and often influenced by multiple phenotypes combining to cause or exacerbate disease. The co-presence of multiple comorbidities and disease sequalae are common in patients who are at higher risk of contracting MRSA infection [58–60]. Interestingly, the comorbidities diabetes and hemodialysis were significantly positively correlated with each other in the APMB group which was not seen in ARMB (Fig. 4). The dual presence of diabetes and dialysis has previously been associated with a higher risk of MRSA compared to MSSA infection [61–63], and this pattern occurred in patients that develop APMB in our study. Diabetes has also been shown to impact the immune response to various infections including *S. aureus* [64–66]. These results suggest that the co-presence of specific clinical phenotypes can impact the canonical cytokine response to infection, leading to a dysfunctional immune response that may promote metastatic seeding and/or *S. aureus* persistence adaptations that in turn perpetuate into APMB outcomes. For example, infective endocarditis yielding cardiac vegetations, another important clinical phenotype in our study, is well known to comprise biofilms in which MRSA small-colony variants develop and may hematogenously disseminate [67]. This could impact the immune response by causing consistent exposure to foreign antigen, increased dysfunctional cytokine production, and an overall phenotype of immune exhaustion similar to that seen in chronic viral infection and sepsis [68, 69]. These findings raise important mechanistic questions on the interplay between pathophysiology of comorbidities, the immune response, and how *S. aureus* may exploit one or more of these issues during infection. Our results suggest that such characteristics should be considered as important co-phenotypes in any clinically applicable prognostic algorithm for evaluating risk as well as optimizing therapeutic outcomes.

Several demographic and clinical characteristics are known to be associated with patient mortality or complications in the course of general MRSA bacteremia [17–19, 36]. These include patient age; sex; presence of prosthetic or indwelling devices; co-morbidities such as immunosuppression, HIV, renal failure requiring hemodialysis, or solid tumors; and possibly race/ethnicity. Some of these parameters are also known to influence cytokine production [70–74]. For these reasons, our cohort was extensively matched on these parameters. Despite our extensive matching, we found that metastatic infection and cardiac vegetation were significantly associated with elevated cytokines in the APMB group, specifically IL-10. This relationship is not unexpected, given that infected cardiac valve vegetations are prone to chronically seed the bloodstream, resulting in metastatic infection and/or septic embolism [75]. Increased IL-10 was positively correlated with both metastatic infection (trending) and cardiac vegetation (significant) in APMB patients, but not in patients with ARMB (Fig. 4). The presence of metastatic infection or cardiac vegetation in MRSA infection is classified as “complicated MRSA infection” according to current clinical guidelines [6]. These patients often require prolonged antibiotic usage to control infection and also commonly succumb to infection, both outcomes of which were seen in our APMB results. The positive correlations between IL-10 and the presence of this specific pair of clinical phenotypes infers a link between immune modulation and more complicated MRSA bacteremia. In turn, this relationship is consistent with our findings of an early dysregulation of the immune response that correlates with slower clearance of bacteria, prolonged bacterial burden, and APMB outcomes. Collectively, our study reveals a complex relationship between cytokine response to MRSA bacteremia and existing clinical phenotypes, and it offers a combinatorial approach integrating cytokine and chemokine responses with clinical phenotypes to gain insights into development of APMB. Future clinical validation of such a composite biomarker and phenotypic signature holds promise to inform early treatment strategies especially in high-risk individuals, such as those with infective endocarditis, metastatic sequelae, and chronic or systemic comorbidities.

### Study limitations

The patient cohort was derived from a single academic medical center and was smaller than multi-center studies, potentially creating a geographic bias associated with demography. Additionally, there was a lack of longitudinal sampling for this patient cohort which limits information available on the evolution of the cytokine response, specifically the transition from innate to adaptive immunity. Only patients who received initial VAN therapy were selected for this study, which limits interpretation of our findings to this antibiotic. Future studies are necessary to assess whether similar relationships exist in the setting of other antibiotic regimens. Beyond the scope of the current study, our ongoing experimental models in vitro and in vivo are comparing outcomes in the context of distinct antibiotic therapy. Indeed, we anticipate that persistent vs. resolving outcomes will differ relative to therapies, and we hypothesize these differences will reflect distinct immune response profiles.

Our findings may be confounded by potential differences in collection periods for each individual cohort, when different strains of MRSA could have been present and could have impacted the cytokine response leading to increased variability seen in results. The time between onset of bacteremia and the start of antibiotic therapy was also unknown in this patient cohort. The overall bacterial burden was not known for this study, making it difficult to determine causal vs. non-causal correlations among circulating analytes, specific clinical phenotypes such as metastatic infection, and MRSA burden. Previous work has shown the effect of environmental and non-genetic host phenotypes on cytokine responses to multiple stimuli including *S. aureus* [76]. However, neither these prior studies nor the current study addresses potential differences in host response between MRSA and MSSA. However, a recent study by Thaden et al. [77] supports our hypothesis that immune responses to MRSA and MSSA have significant differences and may be influenced by antibiotic regimen and susceptibility. It should be noted that our study investigates only a portion of clinical variables that could influence cytokine responses to MRSA infection and APMB outcomes. Future studies could investigate additional clinical variables that may also impact cytokine responses and infection outcome in the context of specific host phenotypes. Such studies may help identify potential alternate influences behind the other elevated analytes seen in fatal APMB in our study cohort (Supplemental Fig. 4) that were not linked to the clinical phenotypes examined. Finally, on a technical level it should be noted that Luminex assays have limits of detection for each analyte measured, which could skew some of our cytokine/chemokine results and CART analysis if certain analytes were approaching this limit of detection.

## Conclusions

Overall, the current findings identify cytokine/chemokine signatures that discriminate APMB from ARMB. Furthermore, modeling suggests increased accuracy in predicting APMB outcome when using patterns of these analytes in conjunction with specific clinical phenotypes that are associated with more severe SAB. These relationships differentiate APMB from ARMB and lay potential groundwork for a novel time- and cost- effective translational method to forecast persistence at time of presentation and diagnosis of MRSA bacteremia. Validating these outcome signatures and elucidating their host-pathogen relationships holds promise for development of strategies for early identification of patients at high risk of developing APMB. Such a predictive capability could inform interventions to improve anti-infective regimens or strategies to mitigate the risks of persistence which is often associated with severe outcomes.

## Supplementary Information

Supplementary Material 1

Supplementary Material 2

Supplementary Material 3

## Data Availability

The datasets used and/or analyzed during the current study are available from the corresponding author(s) on reasonable request.
