# Supplementary material for: Early cytokine signatures and clinical phenotypes discriminate persistent from resolving MRSA bacteremia
Source: BMC Infect Dis. 2025 Feb 18;25:231. doi: 10.1186/s12879-025-10620-3 (PMC11834594; doi:10.1186/s12879-025-10620-3)
Supplement: Supplementary file 1 — Supplementary Material 1 [file 12879_2025_10620_MOESM1_ESM.pdf]

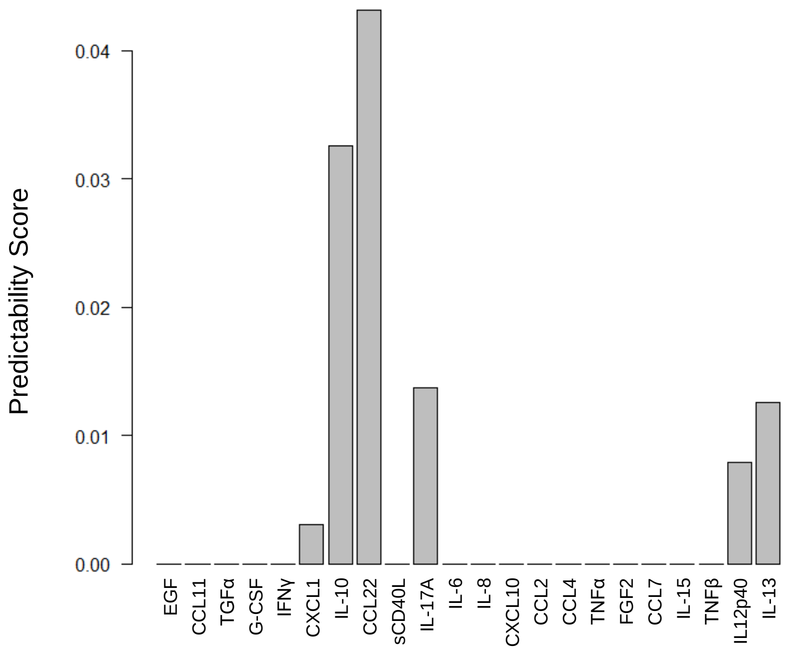

Supplemental Figure 1: Lasso regression model for 22 analytes from Figure 1. X-axis shows individual cytokines and chemokines, Y-axis shows score depicting “importance” in predicting infection outcome. “Predictive” analytes identified as y>0.00.

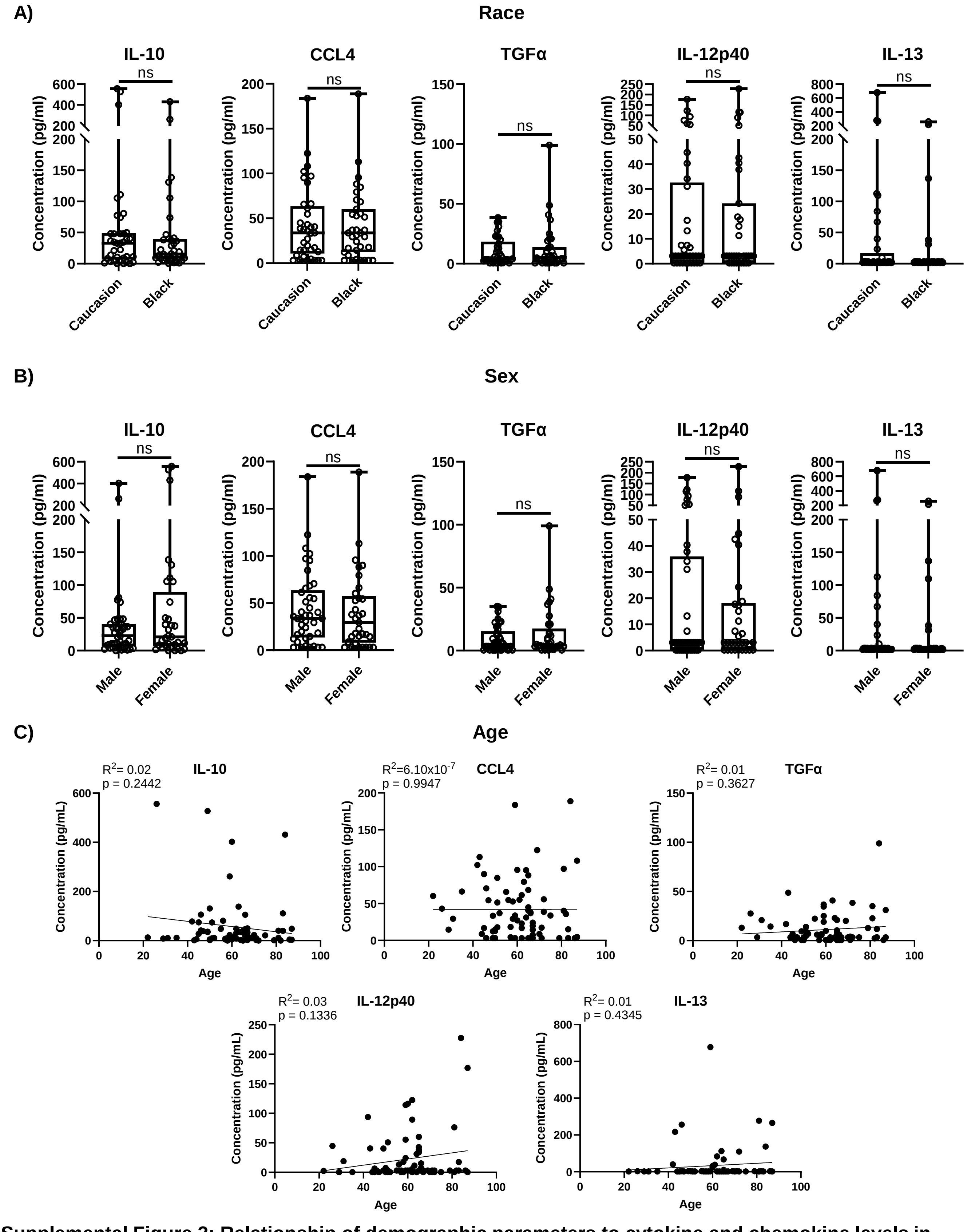

Supplemental Figure 2: Relationship of demographic parameters to cytokine and chemokine levels in APMB. Cytokines and chemokines from Figure 2 were analyzed in the APMB group based on race **(A)**, sex **(B)**, and age **(C)**. Significance determined via two-way unpaired t-test (A-B) or simple linear regression (C).

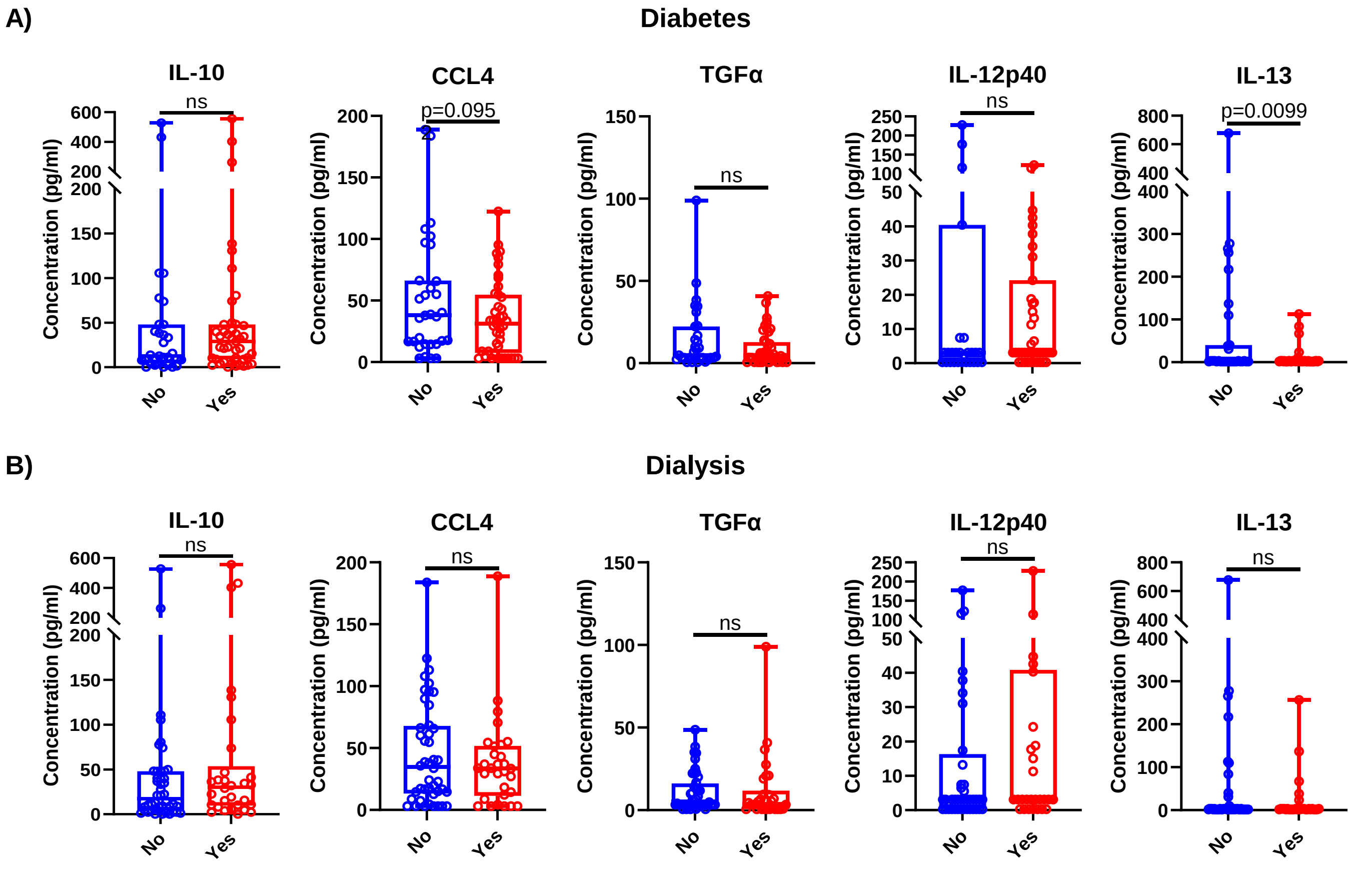

Supplemental Figure 3: Relationship of underlying conditions to cytokine and chemokine levels in APMB. Cytokines/chemokines from Figure 2 were analyzed based on presence or absence of **A)** diabetes and **B)** dialysis as underlying conditions in the APMB patient group. Significance determined via two-way unpaired t-test.

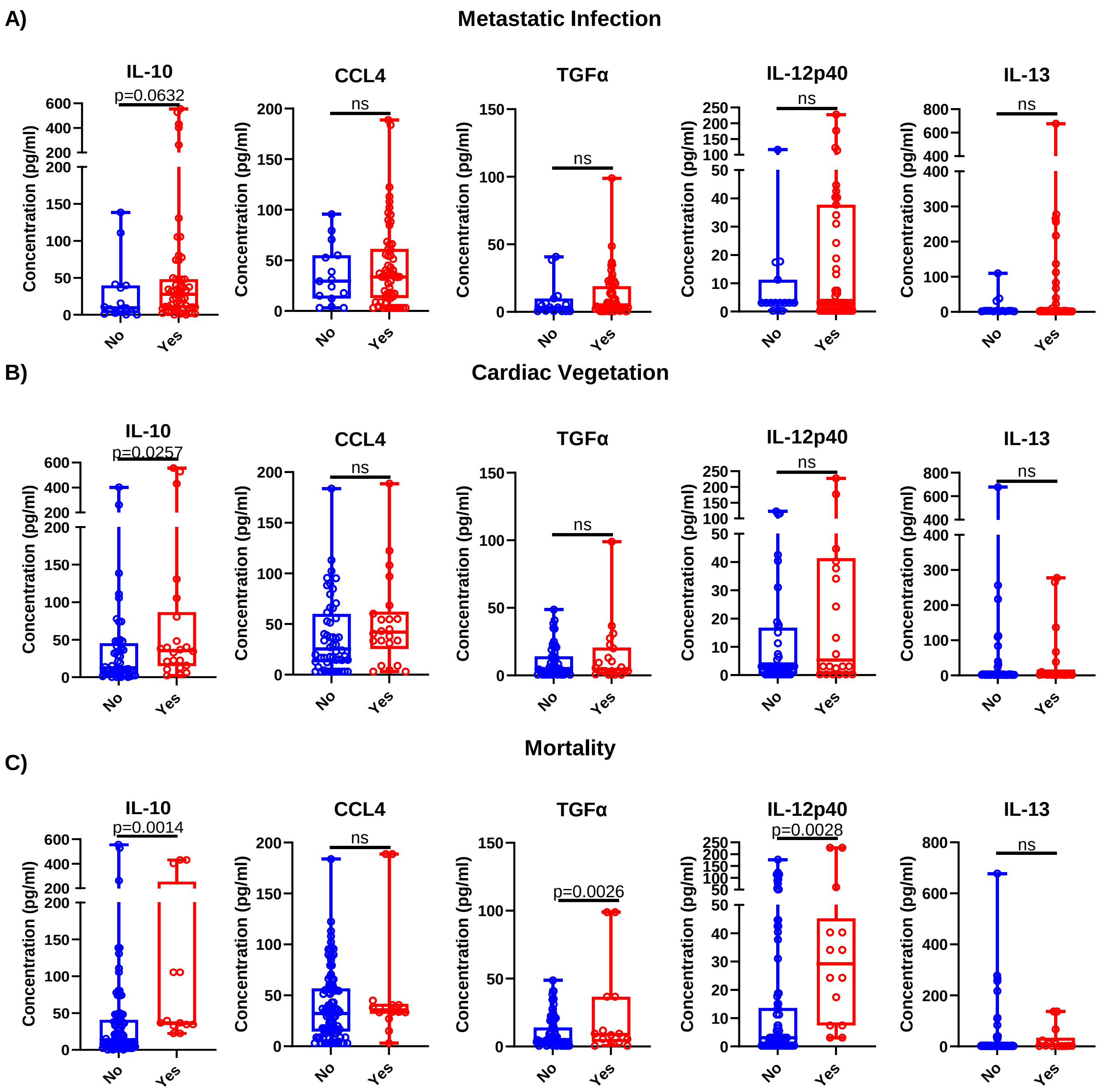

Supplemental Figure 4: Relationship of individual clinical phenotypes and overall mortality to cytokine and chemokine levels in APMB. Cytokines/chemokines from Figure 2 were analyzed in the APMB group based on presence or absence of the following clinical variables: **A)** metastatic infection, **B)** cardiac vegetation, and **C)** all-cause mortality in the APMB patient group. Significance determined via two-way unpaired t-test.

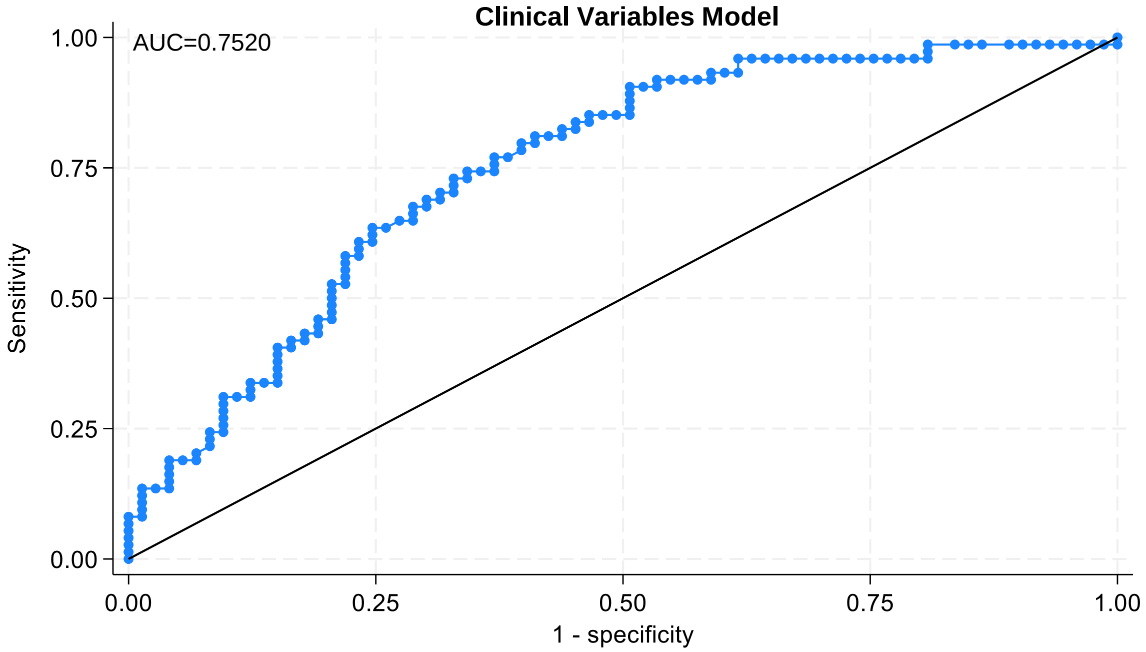

Supplemental Figure 5: Effect of 4 clinical phenotype model on discriminating APMB outcome. Logistic regression model for combined training cohort. ROC curve built using 4 clinical variables.

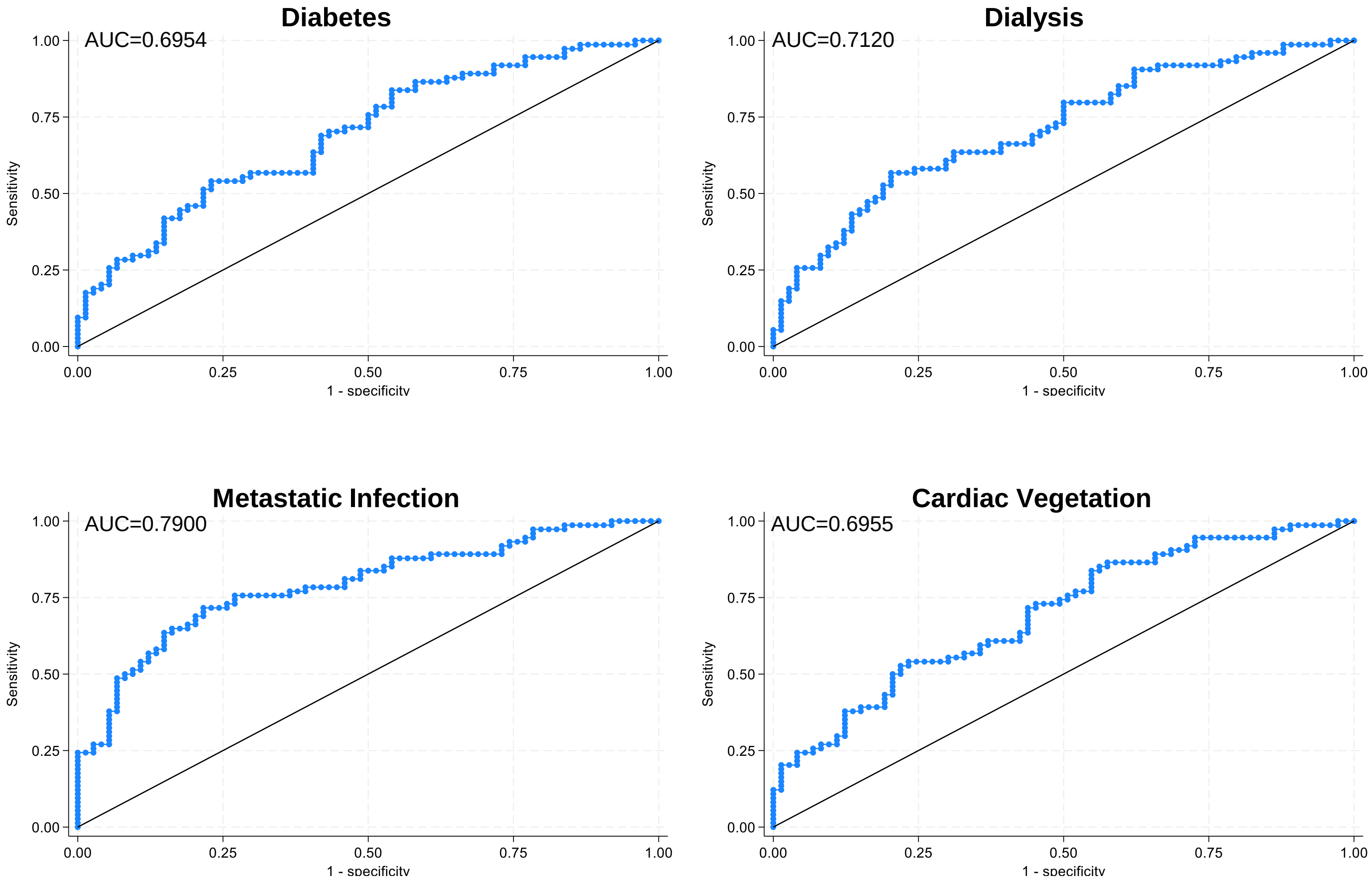

Supplemental Figure 6: Effect of single clinical phenotype inclusion in cytokine model on discriminating APMB outcome. Logistic regression model for combined training cohort. ROC curves built using 8 cytokines/chemokines and single clinical variable. Clinical variable added is shown in title.
