# Supplementary material for: Early cytokine signatures and clinical phenotypes discriminate persistent from resolving MRSA bacteremia
Source: BMC Infect Dis. 2025 Feb 18;25:231. doi: 10.1186/s12879-025-10620-3 (PMC11834594; doi:10.1186/s12879-025-10620-3)
Supplement: Supplementary file 2 — Supplementary Material 2 [file 12879_2025_10620_MOESM2_ESM.docx]

**Supplementary Tables**

**Supplementary Table 2. Prediction modeling for validation cohort, without cardiac vegetation parameter, using plasma samples only*.**

| **Parameter** | **ARMB and APMB**  **(n=77)** | **ARMB Only**  **(n=54)** | **APMB**  **Only**  **(n=23)** |
| --- | --- | --- | --- |
| Outcomes Accurately Predicted | 55 | 38 | 17 |
|  |  |  |  |
| Sensitivity (%) | 73.9 | 51.6 | 89.8 |
| Specificity (%) | 70.4 | 56.4 | 82.0 |
| ROC Area | 0.721 | 0.611 | 0.832 |
|  |  |  |  |
| Likelihood ratio (+) | 2.49 | 1.55 | 4.02 |
| Likelihood ratio (-) | 0.371 | 0.182 | 0.754 |
| Odds Ratio | 6.73 | 2.29 | 19.70 |
|  |  |  |  |
| Positive Predictive Value (%) | 71.4 | 60.7 | 80.1 |
| Negative Predictive Value (%) | 73.0 | 57.0 | 84.6 |
| Overall Prediction Accuracy = (38+17)/77 = 71% | | | |

*Values determined using 95% confidence interval.

**Supplementary Table 3.** **Prediction modeling for blinded validation cohort, without cardiac vegetation parameter, using serum samples only*.**

| **Parameter** | **ARMB and APMB**  **(n=67)** | **ARMB Only**  **(n=41)** | **APMB**  **Only**  **(n=26)** |
| --- | --- | --- | --- |
| Outcomes Accurately Predicted | 40 | 36 | 4 |
|  |  |  |  |
| Sensitivity (%) | 15.4 | 4.36 | 34.9 |
| Specificity (%) | 87.8 | 73.8 | 95.9 |
| ROC Area | 0.516 | 0.429 | 0.603 |
|  |  |  |  |
| Likelihood ratio (+) | 1.26 | 0.373 | 4.27 |
| Likelihood ratio (-) | 0.964 | 0.789 | 1.18 |
| Odds Ratio | 1.31 | 0.342 | 5.05 |
|  |  |  |  |
| Positive Predictive Value (%) | 55.8 | 27.1 | 81.0 |
| Negative Predictive Value (%) | 50.9 | 45.9 | 55.9 |
| Overall Prediction Accuracy = (36+4)/67 = 59.7% | | | |

*Values determined using 95% confidence interval.
